# *n*-Undeca­nyl 2-(2,4-dichloro­anilino)-4,4-dimethyl-6-oxocyclo­hex-1-ene­carbo­dithio­ate

**DOI:** 10.1107/S1600536809006187

**Published:** 2009-02-25

**Authors:** El Sayed H. El Ashry, Mohammed R. Amer, M. Raza Shah, Seik Weng Ng

**Affiliations:** a H.E.J. Research Institute of Chemistry, International Center for Chemical and Biological Sciences, University of Karachi, Karachi 75270, Pakistan; b Department of Chemistry, University of Malaya, 50603 Kuala Lumpur, Malaysia

## Abstract

The six-membered cyclo­hexene ring in the title compound, C_26_H_37_Cl_2_NOS_2_, adopts an envelope-shaped conformation, with the C atom bearing the two methyl groups representing the flap. This atom deviates by 0.658 (7) Å from the plane passing through the other five atoms of the ring (r.m.s. deviation = 0.005 Å). The mol­ecular conformation is stabilized by an N—H⋯S hydrogen bond.

## Related literature

For background, see: El Ashry *et al.* (2009 ▶).
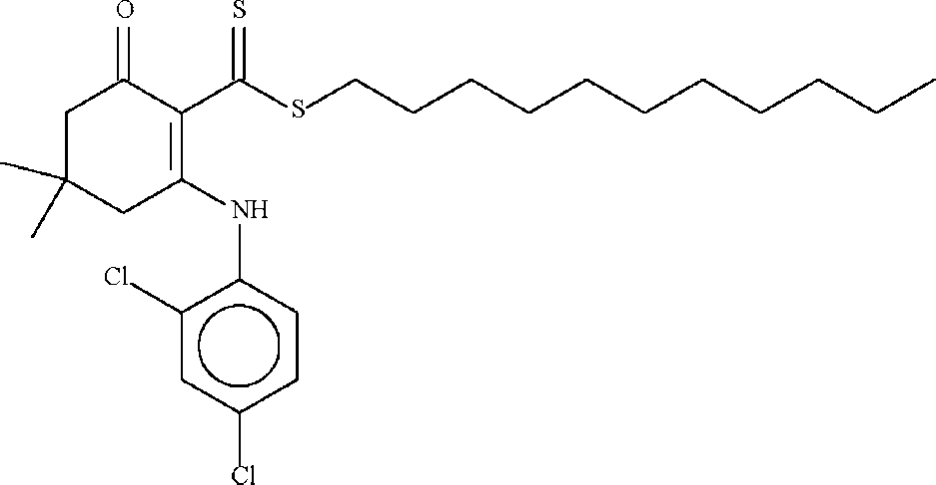


## Experimental

### 

#### Crystal data

C_26_H_37_Cl_2_NOS_2_
*M* *_r_* = 514.59
Orthorhombic, 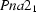

*a* = 14.6145 (4) Å
*b* = 31.378 (1) Å
*c* = 5.9332 (2) Å
*V* = 2720.8 (2) Å^3^
*Z* = 4
Mo *K*α radiation
μ = 0.41 mm^−1^
*T* = 100 K
0.30 × 0.20 × 0.02 mm

#### Data collection

Bruker SMART APEX diffractometer
Absorption correction: multi-scan (*SADABS*; Sheldrick, 1996 ▶) *T*_min_ = 0.665, *T*_max_ = 0.992
24605 measured reflections
6244 independent reflections
4130 reflections with *I* > 2σ(*I*)
*R*_int_ = 0.084

#### Refinement

*R*[*F* ^2^ > 2σ(*F* ^2^)] = 0.064
*wR*(*F* ^2^) = 0.177
*S* = 1.05
6244 reflections
293 parameters
2 restraints
H atoms treated by a mixture of independent and constrained refinement
Δρ_max_ = 0.37 e Å^−3^
Δρ_min_ = −0.40 e Å^−3^
Absolute structure: Flack (1983 ▶), 2816 Friedel pairs
Flack parameter: 0.1 (1)

### 

Data collection: *APEX2* (Bruker, 2008 ▶); cell refinement: *SAINT* (Bruker, 2008 ▶); data reduction: *SAINT*; program(s) used to solve structure: *SHELXS97* (Sheldrick, 2008 ▶); program(s) used to refine structure: *SHELXL97* (Sheldrick, 2008 ▶); molecular graphics: *X-SEED* (Barbour, 2001 ▶); software used to prepare material for publication: *publCIF* (Westrip, 2009 ▶).

## Supplementary Material

Crystal structure: contains datablocks global, I. DOI: 10.1107/S1600536809006187/bt2880sup1.cif

Structure factors: contains datablocks I. DOI: 10.1107/S1600536809006187/bt2880Isup2.hkl

Additional supplementary materials:  crystallographic information; 3D view; checkCIF report

## Figures and Tables

**Table 1:** Hydrogen-bond geometry (Å, °)

*D*—H⋯*A*	*D*—H	H⋯*A*	*D*⋯*A*	*D*—H⋯*A*
N1—H1⋯S2	0.88 (1)	2.09 (3)	2.867 (4)	147 (5)

**Fig. 1.**
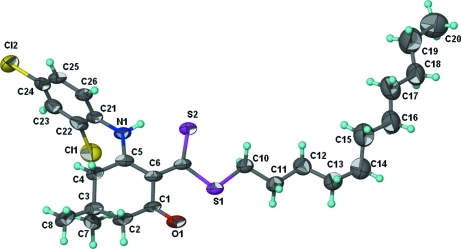
Anisotropic displacement ellipsoid plot (Barbour, 2001) plot of C26H37Cl2NOS2 at the 70% probability level; hydrogen atoms are drawn as spheres of arbitrary radius.

## supplementary crystallographic information

### Experimental

To a solution of (2,4-dichlorophenylamino)-5,5-dimethyl-cyclohex-2-en-1-one (0.1 mol) in DMSO (20 ml) and sodium hydroxide (0.4 g) in water (1 ml), carbon disulfphide (0.3 mol) was added in the course of 30 minutes. The mixture was stirred for 20 min at 283 K, and then 1-bromoundecane (0.1 mol) was added drop wise at room temperature for 30 min. The reaction mixture was left for 24 h and then diluted with water (200 ml) and acidified with 10% hydrochloric acid. The resulting precipitate was collected by filtration, dried and purified on silica gel column (40% ethyl acetate in hexane) to give yellow crystal (31% yield; mp.350 K).

### Refinement

Carbon-bound H-atoms were placed in calculated positions (C—H 0.93 to 0.99 Å) and were included in the refinement in the riding model approximation, with *U*(H) set to 1.2 to 1.5*U*(C).

The amino H-atom was located in a difference Fourier map, and was refined with a distance restraint of N–H 0.88±0.01 Å; its isotropic displacement parameter was freely refined.

### Crystal data

**Table:**

C_26_H_37_Cl_2_NOS_2_	*F*(000) = 1096
*M**_r_* = 514.59	*D*_x_ = 1.256 Mg m^−^^3^
Orthorhombic, *P**n**a*2_1_	Mo *K*α radiation, λ = 0.71073 Å
Hall symbol: P 2c -2n	Cell parameters from 2890 reflections
*a* = 14.6145 (4) Å	θ = 2.6–19.4°
*b* = 31.378 (1) Å	µ = 0.41 mm^−^^1^
*c* = 5.9332 (2) Å	*T* = 100 K
*V* = 2720.8 (2) Å^3^	Plate, yellow
*Z* = 4	0.30 × 0.20 × 0.02 mm

### Data collection

**Table:**

Bruker SMART APEX diffractometer	6244 independent reflections
Radiation source: fine-focus sealed tube	4130 reflections with *I* > 2σ(*I*)
graphite	*R*_int_ = 0.084
ω scans	θ_max_ = 27.5°, θ_min_ = 1.3°
Absorption correction: multi-scan (*SADABS*; Sheldrick, 1996)	*h* = −18→18
*T*_min_ = 0.665, *T*_max_ = 0.992	*k* = −40→40
24605 measured reflections	*l* = −7→7

### Refinement

**Table:**

Refinement on *F*^2^	Secondary atom site location: difference Fourier map
Least-squares matrix: full	Hydrogen site location: inferred from neighbouring sites
*R*[*F*^2^ > 2σ(*F*^2^)] = 0.064	H atoms treated by a mixture of independent and constrained refinement
*wR*(*F*^2^) = 0.177	*w* = 1/[σ^2^(*F*_o_^2^) + (0.09*P*)^2^ + 0.1294*P*] where *P* = (*F*_o_^2^ + 2*F*_c_^2^)/3
*S* = 1.05	(Δ/σ)_max_ = 0.001
6244 reflections	Δρ_max_ = 0.37 e Å^−^^3^
293 parameters	Δρ_min_ = −0.40 e Å^−^^3^
2 restraints	Absolute structure: Flack (1983), 2816 Friedel pairs
Primary atom site location: structure-invariant direct methods	Flack parameter: 0.1 (1)

### Fractional atomic coordinates and isotropic or equivalent isotropic displacement parameters (Å^2^)

**Table:**

	*x*	*y*	*z*	*U*_iso_*/*U*_eq_	
Cl1	1.00959 (9)	0.61556 (4)	0.0000 (3)	0.0572 (4)	
Cl2	0.78356 (10)	0.56460 (5)	0.6578 (3)	0.0792 (6)	
S1	1.14467 (7)	0.80008 (4)	−0.2529 (2)	0.0428 (3)	
S2	0.96442 (7)	0.76693 (4)	−0.1253 (2)	0.0438 (3)	
O1	1.2659 (2)	0.77730 (11)	0.0313 (7)	0.0582 (11)	
N1	0.9918 (2)	0.70203 (12)	0.2082 (7)	0.0368 (9)	
H1	0.961 (3)	0.7208 (14)	0.128 (9)	0.072 (19)*	
C1	1.2230 (3)	0.75073 (15)	0.1403 (9)	0.0398 (11)	
C2	1.2713 (3)	0.72670 (17)	0.3218 (8)	0.0461 (13)	
H2A	1.2694	0.7439	0.4616	0.055*	
H2B	1.3363	0.7235	0.2782	0.055*	
C3	1.2328 (3)	0.68261 (15)	0.3737 (8)	0.0388 (10)	
C4	1.1304 (3)	0.68848 (14)	0.4192 (7)	0.0354 (10)	
H4A	1.1019	0.6601	0.4382	0.042*	
H4B	1.1227	0.7042	0.5626	0.042*	
C5	1.0805 (3)	0.71211 (13)	0.2352 (8)	0.0342 (9)	
C6	1.1242 (3)	0.74336 (14)	0.0975 (8)	0.0358 (10)	
C7	1.2483 (3)	0.65146 (16)	0.1819 (8)	0.0461 (12)	
H7A	1.3138	0.6496	0.1490	0.069*	
H7B	1.2156	0.6614	0.0475	0.069*	
H7C	1.2253	0.6233	0.2252	0.069*	
C8	1.2777 (3)	0.6654 (2)	0.5899 (8)	0.0539 (14)	
H8A	1.3432	0.6609	0.5636	0.081*	
H8B	1.2490	0.6383	0.6315	0.081*	
H8C	1.2694	0.6860	0.7123	0.081*	
C9	1.0777 (3)	0.76748 (13)	−0.0756 (8)	0.0345 (10)	
C10	1.0630 (3)	0.82146 (14)	−0.4492 (8)	0.0410 (11)	
H10A	1.0362	0.7982	−0.5407	0.049*	
H10B	1.0130	0.8360	−0.3672	0.049*	
C11	1.1127 (3)	0.85293 (15)	−0.6007 (9)	0.0440 (11)	
H11A	1.1425	0.8749	−0.5059	0.053*	
H11B	1.1613	0.8377	−0.6847	0.053*	
C12	1.0492 (3)	0.87471 (15)	−0.7678 (10)	0.0478 (12)	
H12A	1.0284	0.8535	−0.8801	0.057*	
H12B	0.9946	0.8852	−0.6864	0.057*	
C13	1.0937 (4)	0.91173 (17)	−0.8903 (10)	0.0566 (14)	
H13A	1.1036	0.9351	−0.7807	0.068*	
H13B	1.1546	0.9024	−0.9443	0.068*	
C14	1.0409 (4)	0.9295 (2)	−1.0893 (11)	0.0703 (17)	
H14A	1.0727	0.9552	−1.1457	0.084*	
H14B	1.0409	0.9081	−1.2119	0.084*	
C15	0.9460 (5)	0.9406 (2)	−1.0351 (12)	0.0747 (18)	
H15A	0.9457	0.9636	−0.9209	0.090*	
H15B	0.9152	0.9155	−0.9687	0.090*	
C16	0.8908 (4)	0.9558 (2)	−1.2488 (13)	0.0769 (19)	
H16A	0.9145	0.9838	−1.2989	0.092*	
H16B	0.9001	0.9352	−1.3730	0.092*	
C17	0.7924 (5)	0.9595 (2)	−1.2005 (12)	0.0746 (19)	
H17A	0.7838	0.9807	−1.0786	0.090*	
H17B	0.7700	0.9317	−1.1441	0.090*	
C18	0.7344 (4)	0.9726 (2)	−1.4016 (13)	0.078 (2)	
H18A	0.7475	1.0028	−1.4396	0.093*	
H18B	0.7512	0.9549	−1.5334	0.093*	
C19	0.6346 (5)	0.9677 (3)	−1.3545 (15)	0.097 (2)	
H19A	0.6222	0.9374	−1.3190	0.117*	
H19B	0.6191	0.9846	−1.2191	0.117*	
C20	0.5735 (5)	0.9809 (3)	−1.5409 (15)	0.103 (3)	
H20A	0.5099	0.9744	−1.5012	0.155*	
H20B	0.5902	0.9655	−1.6785	0.155*	
H20C	0.5799	1.0116	−1.5663	0.155*	
C21	0.9445 (3)	0.66872 (13)	0.3226 (8)	0.0363 (11)	
C22	0.9440 (3)	0.62774 (14)	0.2343 (10)	0.0420 (11)	
C23	0.8932 (3)	0.59622 (15)	0.3351 (9)	0.0448 (12)	
H23	0.8915	0.5685	0.2712	0.054*	
C24	0.8448 (3)	0.60482 (16)	0.5284 (11)	0.0511 (14)	
C25	0.8451 (3)	0.64514 (16)	0.6211 (10)	0.0491 (13)	
H25	0.8117	0.6509	0.7550	0.059*	
C26	0.8953 (3)	0.67727 (14)	0.5153 (9)	0.0408 (11)	
H26	0.8955	0.7052	0.5767	0.049*	

### Atomic displacement parameters (Å^2^)

**Table:**

	*U*^11^	*U*^22^	*U*^33^	*U*^12^	*U*^13^	*U*^23^
Cl1	0.0577 (8)	0.0573 (8)	0.0566 (7)	0.0050 (6)	0.0064 (7)	−0.0103 (7)
Cl2	0.0463 (8)	0.0621 (9)	0.1293 (15)	−0.0022 (7)	0.0212 (9)	0.0387 (10)
S1	0.0261 (5)	0.0416 (6)	0.0608 (7)	0.0032 (5)	0.0118 (6)	0.0043 (6)
S2	0.0229 (5)	0.0495 (7)	0.0592 (7)	−0.0022 (5)	0.0030 (6)	0.0071 (6)
O1	0.0199 (15)	0.059 (2)	0.095 (3)	−0.0036 (14)	0.0063 (18)	0.017 (2)
N1	0.0203 (16)	0.039 (2)	0.051 (3)	−0.0035 (15)	0.0032 (17)	−0.0007 (19)
C1	0.0199 (19)	0.045 (2)	0.054 (3)	−0.0005 (19)	0.012 (2)	0.003 (2)
C2	0.022 (2)	0.068 (3)	0.048 (3)	−0.007 (2)	0.0052 (19)	0.000 (2)
C3	0.026 (2)	0.061 (3)	0.030 (2)	−0.0031 (19)	−0.001 (2)	−0.005 (2)
C4	0.026 (2)	0.049 (3)	0.031 (2)	−0.0050 (18)	0.0022 (17)	−0.003 (2)
C5	0.0222 (18)	0.038 (2)	0.043 (2)	0.0000 (16)	0.004 (2)	−0.012 (2)
C6	0.0210 (19)	0.039 (2)	0.048 (3)	0.0015 (17)	0.0090 (19)	−0.007 (2)
C7	0.036 (2)	0.059 (3)	0.043 (3)	0.013 (2)	0.002 (2)	0.006 (2)
C8	0.034 (3)	0.098 (4)	0.030 (2)	−0.017 (3)	−0.004 (2)	0.014 (3)
C9	0.0211 (19)	0.036 (2)	0.047 (3)	−0.0005 (17)	0.0100 (18)	−0.007 (2)
C10	0.033 (2)	0.039 (2)	0.051 (3)	0.0016 (19)	0.008 (2)	0.001 (2)
C11	0.040 (2)	0.043 (3)	0.050 (3)	0.001 (2)	0.013 (2)	0.000 (2)
C12	0.048 (3)	0.047 (3)	0.049 (3)	0.005 (2)	0.001 (3)	−0.002 (2)
C13	0.050 (3)	0.057 (3)	0.063 (3)	0.003 (3)	0.010 (3)	0.008 (3)
C14	0.070 (4)	0.070 (4)	0.071 (4)	0.003 (3)	0.005 (4)	0.008 (3)
C15	0.079 (5)	0.069 (4)	0.076 (4)	0.001 (3)	−0.002 (4)	0.009 (3)
C16	0.069 (4)	0.071 (4)	0.091 (5)	−0.004 (3)	−0.015 (4)	0.026 (4)
C17	0.078 (5)	0.059 (4)	0.087 (5)	0.006 (3)	−0.012 (4)	0.007 (3)
C18	0.063 (4)	0.079 (4)	0.091 (5)	−0.005 (3)	−0.015 (4)	0.031 (4)
C19	0.089 (5)	0.104 (5)	0.099 (6)	−0.018 (4)	−0.015 (5)	0.026 (5)
C20	0.070 (4)	0.125 (6)	0.116 (7)	−0.027 (4)	−0.029 (5)	0.004 (6)
C21	0.0192 (19)	0.039 (2)	0.050 (3)	−0.0032 (17)	−0.0009 (18)	0.003 (2)
C22	0.027 (2)	0.040 (2)	0.059 (3)	0.0040 (18)	−0.008 (2)	−0.005 (2)
C23	0.028 (2)	0.037 (3)	0.069 (4)	−0.0013 (19)	−0.009 (2)	0.002 (2)
C24	0.022 (2)	0.048 (3)	0.084 (4)	0.0005 (19)	−0.002 (2)	0.022 (3)
C25	0.026 (2)	0.057 (3)	0.065 (3)	0.007 (2)	0.017 (2)	0.011 (3)
C26	0.028 (2)	0.036 (2)	0.058 (3)	0.0047 (18)	0.013 (2)	0.000 (2)

### Geometric parameters (Å, °)

**Table:**

Cl1—C22	1.731 (5)	C12—H12A	0.9900
Cl2—C24	1.727 (5)	C12—H12B	0.9900
S1—C9	1.764 (4)	C13—C14	1.516 (8)
S1—C10	1.797 (5)	C13—H13A	0.9900
S2—C9	1.682 (4)	C13—H13B	0.9900
O1—C1	1.228 (5)	C14—C15	1.466 (9)
N1—C5	1.343 (5)	C14—H14A	0.9900
N1—C21	1.425 (6)	C14—H14B	0.9900
N1—H1	0.882 (10)	C15—C16	1.577 (9)
C1—C6	1.483 (6)	C15—H15A	0.9900
C1—C2	1.492 (7)	C15—H15B	0.9900
C2—C3	1.524 (7)	C16—C17	1.470 (8)
C2—H2A	0.9900	C16—H16A	0.9900
C2—H2B	0.9900	C16—H16B	0.9900
C3—C7	1.517 (7)	C17—C18	1.521 (9)
C3—C4	1.533 (6)	C17—H17A	0.9900
C3—C8	1.539 (7)	C17—H17B	0.9900
C4—C5	1.508 (6)	C18—C19	1.494 (9)
C4—H4A	0.9900	C18—H18A	0.9900
C4—H4B	0.9900	C18—H18B	0.9900
C5—C6	1.427 (6)	C19—C20	1.480 (10)
C6—C9	1.446 (7)	C19—H19A	0.9900
C7—H7A	0.9800	C19—H19B	0.9900
C7—H7B	0.9800	C20—H20A	0.9800
C7—H7C	0.9800	C20—H20B	0.9800
C8—H8A	0.9800	C20—H20C	0.9800
C8—H8B	0.9800	C21—C26	1.377 (7)
C8—H8C	0.9800	C21—C22	1.388 (6)
C10—C11	1.520 (6)	C22—C23	1.374 (7)
C10—H10A	0.9900	C23—C24	1.374 (8)
C10—H10B	0.9900	C23—H23	0.9500
C11—C12	1.520 (7)	C24—C25	1.380 (7)
C11—H11A	0.9900	C25—C26	1.395 (6)
C11—H11B	0.9900	C25—H25	0.9500
C12—C13	1.517 (7)	C26—H26	0.9500
			
C9—S1—C10	103.6 (2)	C14—C13—H13A	108.3
C5—N1—C21	125.7 (4)	C12—C13—H13A	108.3
C5—N1—H1	114 (4)	C14—C13—H13B	108.3
C21—N1—H1	120 (4)	C12—C13—H13B	108.3
O1—C1—C6	120.9 (4)	H13A—C13—H13B	107.4
O1—C1—C2	118.8 (4)	C15—C14—C13	113.5 (5)
C6—C1—C2	120.3 (4)	C15—C14—H14A	108.9
C1—C2—C3	115.5 (4)	C13—C14—H14A	108.9
C1—C2—H2A	108.4	C15—C14—H14B	108.9
C3—C2—H2A	108.4	C13—C14—H14B	108.9
C1—C2—H2B	108.4	H14A—C14—H14B	107.7
C3—C2—H2B	108.4	C14—C15—C16	112.3 (6)
H2A—C2—H2B	107.5	C14—C15—H15A	109.1
C7—C3—C2	112.2 (4)	C16—C15—H15A	109.1
C7—C3—C4	110.8 (4)	C14—C15—H15B	109.1
C2—C3—C4	106.7 (4)	C16—C15—H15B	109.1
C7—C3—C8	109.6 (4)	H15A—C15—H15B	107.9
C2—C3—C8	109.3 (4)	C17—C16—C15	111.5 (6)
C4—C3—C8	108.1 (4)	C17—C16—H16A	109.3
C5—C4—C3	113.8 (4)	C15—C16—H16A	109.3
C5—C4—H4A	108.8	C17—C16—H16B	109.3
C3—C4—H4A	108.8	C15—C16—H16B	109.3
C5—C4—H4B	108.8	H16A—C16—H16B	108.0
C3—C4—H4B	108.8	C16—C17—C18	114.4 (6)
H4A—C4—H4B	107.7	C16—C17—H17A	108.7
N1—C5—C6	121.7 (4)	C18—C17—H17A	108.7
N1—C5—C4	115.9 (4)	C16—C17—H17B	108.7
C6—C5—C4	122.4 (4)	C18—C17—H17B	108.7
C5—C6—C9	123.7 (4)	H17A—C17—H17B	107.6
C5—C6—C1	116.4 (4)	C19—C18—C17	111.7 (6)
C9—C6—C1	119.8 (4)	C19—C18—H18A	109.3
C3—C7—H7A	109.5	C17—C18—H18A	109.3
C3—C7—H7B	109.5	C19—C18—H18B	109.3
H7A—C7—H7B	109.5	C17—C18—H18B	109.3
C3—C7—H7C	109.5	H18A—C18—H18B	107.9
H7A—C7—H7C	109.5	C20—C19—C18	114.8 (7)
H7B—C7—H7C	109.5	C20—C19—H19A	108.6
C3—C8—H8A	109.5	C18—C19—H19A	108.6
C3—C8—H8B	109.5	C20—C19—H19B	108.6
H8A—C8—H8B	109.5	C18—C19—H19B	108.6
C3—C8—H8C	109.5	H19A—C19—H19B	107.5
H8A—C8—H8C	109.5	C19—C20—H20A	109.5
H8B—C8—H8C	109.5	C19—C20—H20B	109.5
C6—C9—S2	125.6 (3)	H20A—C20—H20B	109.5
C6—C9—S1	117.8 (3)	C19—C20—H20C	109.5
S2—C9—S1	116.6 (3)	H20A—C20—H20C	109.5
C11—C10—S1	108.0 (3)	H20B—C20—H20C	109.5
C11—C10—H10A	110.1	C26—C21—C22	119.4 (4)
S1—C10—H10A	110.1	C26—C21—N1	120.4 (4)
C11—C10—H10B	110.1	C22—C21—N1	120.1 (4)
S1—C10—H10B	110.1	C23—C22—C21	120.3 (5)
H10A—C10—H10B	108.4	C23—C22—Cl1	119.3 (4)
C12—C11—C10	112.7 (4)	C21—C22—Cl1	120.3 (4)
C12—C11—H11A	109.0	C22—C23—C24	120.0 (5)
C10—C11—H11A	109.0	C22—C23—H23	120.0
C12—C11—H11B	109.0	C24—C23—H23	120.0
C10—C11—H11B	109.0	C23—C24—C25	120.7 (4)
H11A—C11—H11B	107.8	C23—C24—Cl2	119.6 (4)
C13—C12—C11	113.3 (4)	C25—C24—Cl2	119.7 (4)
C13—C12—H12A	108.9	C24—C25—C26	119.0 (5)
C11—C12—H12A	108.9	C24—C25—H25	120.5
C13—C12—H12B	108.9	C26—C25—H25	120.5
C11—C12—H12B	108.9	C21—C26—C25	120.5 (4)
H12A—C12—H12B	107.7	C21—C26—H26	119.8
C14—C13—C12	115.8 (5)	C25—C26—H26	119.8
			
O1—C1—C2—C3	−152.8 (5)	C9—S1—C10—C11	175.8 (3)
C6—C1—C2—C3	28.9 (6)	S1—C10—C11—C12	−177.4 (3)
C1—C2—C3—C7	68.3 (5)	C10—C11—C12—C13	169.5 (4)
C1—C2—C3—C4	−53.1 (5)	C11—C12—C13—C14	168.9 (5)
C1—C2—C3—C8	−169.8 (4)	C12—C13—C14—C15	52.6 (7)
C7—C3—C4—C5	−69.6 (5)	C13—C14—C15—C16	−175.9 (5)
C2—C3—C4—C5	52.8 (5)	C14—C15—C16—C17	169.8 (6)
C8—C3—C4—C5	170.2 (4)	C15—C16—C17—C18	−178.1 (6)
C21—N1—C5—C6	173.9 (4)	C16—C17—C18—C19	169.0 (6)
C21—N1—C5—C4	−6.2 (6)	C17—C18—C19—C20	178.5 (7)
C3—C4—C5—N1	150.6 (4)	C5—N1—C21—C26	95.2 (5)
C3—C4—C5—C6	−29.5 (6)	C5—N1—C21—C22	−87.9 (6)
N1—C5—C6—C9	1.9 (7)	C26—C21—C22—C23	1.6 (7)
C4—C5—C6—C9	−178.1 (4)	N1—C21—C22—C23	−175.4 (4)
N1—C5—C6—C1	−178.4 (4)	C26—C21—C22—Cl1	−176.9 (3)
C4—C5—C6—C1	1.6 (6)	N1—C21—C22—Cl1	6.1 (6)
O1—C1—C6—C5	−179.3 (4)	C21—C22—C23—C24	−2.1 (7)
C2—C1—C6—C5	−1.0 (6)	Cl1—C22—C23—C24	176.4 (4)
O1—C1—C6—C9	0.4 (7)	C22—C23—C24—C25	1.2 (7)
C2—C1—C6—C9	178.7 (4)	C22—C23—C24—Cl2	−178.3 (4)
C5—C6—C9—S2	6.9 (6)	C23—C24—C25—C26	0.1 (7)
C1—C6—C9—S2	−172.8 (4)	Cl2—C24—C25—C26	179.7 (4)
C5—C6—C9—S1	−172.5 (3)	C22—C21—C26—C25	−0.2 (7)
C1—C6—C9—S1	7.8 (5)	N1—C21—C26—C25	176.8 (4)
C10—S1—C9—C6	176.9 (3)	C24—C25—C26—C21	−0.7 (7)
C10—S1—C9—S2	−2.6 (3)		

### Hydrogen-bond geometry (Å, °)

**Table:**

*D*—H···*A*	*D*—H	H···*A*	*D*···*A*	*D*—H···*A*
N1—H1···S2	0.88 (1)	2.09 (3)	2.867 (4)	147 (5)
